# Parametrized Graph Convolutional Multi-Agent Reinforcement Learning with Hybrid Action Spaces in Dynamic Topologies

**DOI:** 10.3390/biomimetics11040232

**Published:** 2026-04-01

**Authors:** Pei Chi, Chen Liu, Jiang Zhao, Yingxun Wang

**Affiliations:** 1 Institute of Unmanned System, Beihang University, Beijing 100191, China; peichi@buaa.edu.cn (P.C.); wangyx@buaa.edu.cn (Y.W.); 2 School of Automation Science and Electrical Engineering, Beihang University, Beijing 100191, China; chenliu@buaa.edu.cn

**Keywords:** multi-agent reinforcement learning (MARL), dynamic topology, hybrid action spaces, parametrized graph convolution reinforcement learning (P-DGN), multi-head attention mechanism

## Abstract

Multi-agent swarm collaboration, inspired by the collective behaviors of biological swarms in nature, has wide applications in dynamic open environments. However, hybrid action spaces in multi-agent reinforcement learning (MARL) present a critical challenge: the inherent coupling between discrete and continuous actions severely undermines policy stability and convergence, especially under dynamic topologies. Existing methods fail to decouple this coupling, leading to suboptimal policies and unstable training. This paper addresses the core problem of action coupling under dynamic topologies, proposing a Parametrized Graph Convolution Reinforcement Learning (P-DGN) method. Operating within the actor–critic framework, P-DGN decouples the optimization pathways for hybrid actions, with a biomimetic observation design inspired by starling flock behaviors: each agent only observes the states of its seven nearest neighbors to achieve efficient local interaction and global collaboration. Its actor network uses multi-head attention to build dynamic relation kernels, develops temporal relation regularization (TRR) to improve policy consistency across time steps, and generates continuous actions with a Gaussian policy. Meanwhile, P-DGN’s critic network, based on deep Q-network (DQN), evaluates Q-values for discrete actions to guide optimal choices. We evaluate P-DGN in two different multi-agent cooperative environments. Experimental results show that compared with parametrized deep Q-network (P-DQN) and DQN baseline, the proposed method has faster convergence speed and stronger training stability. Moreover, with dense rewards, P-DGN agents learn emergent tactics like encirclement. Overall, P-DGN offers a new approach for optimizing hybrid action spaces in multi-agent systems within open, dynamic environments, balancing theoretical generality with practical utility, and its biomimetic design provides a biologically plausible framework for multi-agent swarm collaboration.

## 1. Introduction

Multi-agent collaborative systems have attracted extensive attention due to their wide applications in unmanned swarm combat, intelligent transportation, and industrial collaborative manufacturing. The core inspiration for multi-agent swarm collaboration comes from biomimetic observations of collective behaviors in natural biological swarms: from the coordinated flight of starling flocks to the foraging of ant colonies, these biological swarms can achieve robust, efficient global collaborative behaviors through simple local interactions between individuals, without centralized control. This biological mechanism provides a natural blueprint for the design of multi-agent collaborative algorithms in dynamic open environments.

Multi-agent reinforcement learning (MARL) algorithms are designed for systems composed of multiple interacting agents (e.g., robots, machines, vehicles) operating within a shared environment [1]. At each time step, every agent makes independent decisions while coordinating with others to achieve their individual predefined objectives. The goal of MARL is to learn optimal policies for each agent that collectively maximize the system’s global long-term discounted cumulative rewards [2]. Agents, as learnable units, learn optimal strategies through real-time interaction with the environment, while training such agents remains challenging due to the complexity of dynamic environments. Many problems addressed by MARL are classified as NP-hard, including manufacturing scheduling [3], vehicle routing [4,5], and certain multi-agent games [6], among others.

Hybrid action space requires agents to simultaneously process discrete decisions (e.g., skill selection) and continuous parameter control (e.g., motion amplitude) [7]. This demand arises ubiquitously in real-world scenarios: autonomous vehicles, for instance, must collaboratively optimize lane-changing (discrete action) and steering-angle adjustment (continuous parameter) [8]. Traditional methods often yield suboptimal policies due to coupled interference between discrete and continuous actions.

This coupling problem is further exacerbated by dynamic topologies, where relationships between mutually cooperating agents and the reliability of their observations evolve continuously. Agents operating in scenarios characterized by abrupt traffic flow disruptions [9] or unmanned aerial vehicle (UAV) formation reconstruction [10] must concurrently address compound challenges encompassing non-stationary reward functions, time-varying topological structures, and degraded reliability of local observations. For instance, continuous topological evolution necessitates algorithmic capabilities for real-time adaptation of inter-agent connectivity [11]. Furthermore, intensified partial observability compels agents to infer global states from constrained information, substantially amplifying policy optimization complexity under dynamic uncertainties [12].

To address the aforementioned challenges, this paper proposes parametrized graph convolutional reinforcement learning (P-DGN), designed to achieve efficient collaboration under complex dynamic topologies. The method employs multi-head attention mechanisms to capture agents’ topological evolution patterns while leveraging actor–critic framework to establish isolated optimization pathways for discrete action selection and continuous parameter adjustment. This dual-stream design effectively mitigates policy interference and enhances environmental adaptability. Compared with existing approaches, P-DGN’s innovations lie in its parametric modeling of dynamic graph convolutional structure and decoupled representation of intrinsic couplings in hybrid action spaces, thereby providing novel insights for multi-agent system (MAS) deployment in open dynamic scenarios.

A core biomimetic design of our proposed P-DGN method lies in the observation space construction. Inspired by the classic research on starling flocks, which found that individual starlings in a large flock only respond to the states of their seven nearest neighbors to achieve low-cost, high-robustness consensus of the whole swarm [13], we design the observation space of each agent to only include the states of its seven nearest neighbors. This design not only reduces the dimension of the observation space and the computational overhead of the algorithm but also improves the adaptability of the algorithm to dynamic swarm topologies, as the local interaction mechanism is consistent with the biological swarm’s ability to maintain collaboration under dynamic changes of individual positions.

In addition, the deep reinforcement learning framework adopted in this work is essentially a biomimetic simulation of biological learning mechanisms: the neural network simulates the information processing ability of the biological nervous system, and the reinforcement learning paradigm simulates the trial-and-error learning process of organisms, which optimize their behaviors through the feedback of rewards from the environment.

Overall, the main contributions of this work are as follows:

(1) We develop a dynamic graph co-sampling mechanism within parameter-sharing framework, enabling explicit modeling of complex multi-agent interactions in dynamic environments. This innovation overcomes representational limitations inherent in conventional parameter-sharing approaches for sophisticated cooperation tasks.
(2) We establish a novel solution paradigm for hybrid action spaces in MARL via the actor–critic framework: The actor network generates continuous parameters per action, while the critic network evaluates Q-values for each parametrized action, enabling P-DGN to select discrete action types through explicit Q-value maximization.
(3) Topological stability constraint and temporal relation regularization (TRR) are devised and applied to stabilize multi-agent learning in dynamic topologies, ensuring substantially smoother policy convergence.

The paper is organized as follows: Section 2 comprehensively reviews and critiques existing research addressing reinforcement learning (RL) in hybrid action spaces and dynamic topologies. Section 3 elaborates the proposed P-DGN algorithm with full architectural details. Our methodology undergoes rigorous experimental validation in Section 4 through comparative benchmarking against state-of-the-art approaches. Section 5 concludes by synthesizing core innovations and contributions.

## 2. Related Work

This section surveys the most pertinent recent research addressing RL within hybrid action spaces and dynamic topologies and clarifies the core limitations of existing methods.

Due to the inherent coupling between discrete and continuous action dimensions in hybrid action spaces, effective MARL requires explicitly addressing interdependencies. Xie et al. [14] introduced multi-agent branching Q-networks (MABQN), an enhanced QMIX architecture integrating action discretization and value decomposition. MABQN narrows the policy search space by progressively discretizing continuous actions and decoupling action dimensions, thereby improving learning efficiency. Tian et al. [15] proposed multi-agent sequential Q-networks (MASQN), applicable to multi-agent domains with continuous, multi-discrete, or hybrid action spaces. Hua et al. [16] developed two novel approaches: multi-agent hybrid deep deterministic policy gradients (MAHDDPGs) and multi-agent hybrid soft actor–critic (MAHSAC), addressing multi-agent problems in discrete-continuous hybrid action spaces through centralized training with decentralized execution (CTDE) frameworks. Li et al. [17] devised structured cooperative reinforcement learning (SCORE) based on centralized critics and decentralized actors, modeling single-agent hybrid action problems as fully cooperative partially observable stochastic games while employing graph attention networks to capture dependencies among heterogeneous sub-actions. Li et al. [18] implemented an integrated attentional eco-driving policy to investigate strategic variations under diverse vehicle interactions. To tackle hybrid action space challenges, these innovations utilize action discretization, sequential decision-making, graph attention mechanisms, or attentional interaction modeling, effectively decoupling heterogeneous action dependencies and enhancing learning efficiency in static scenarios.

Despite the effectiveness of the above methods in static scenarios, they have critical limitations: all these decoupling strategies are designed for fixed communication topologies and do not take into account the impact of dynamic topology changes on the coupling relationship between discrete and continuous actions. When the agent neighbor topology changes dynamically, the original action decoupling mechanism will face severe distribution shift of observation and interaction features, which will easily lead to the failure of decoupling, resulting in policy oscillation, unstable training and even non-convergence.

Confronting MARL in dynamic topologies, prior research predominantly advances through three distinct paradigms: graph neural network (GNN), attention mechanisms, and their hybrid integrations. Bernárdez et al. [19] devised a novel distributed traffic engineering (TE) system leveraging machine learning advancements, implementing an architecture that synergizes MARL with GNN to minimize network congestion. Ding et al. [20] investigated efficient extraction and utilization of neighboring agents’ information within graph structures to derive high-fidelity feature representations for collaborative tasks. Wang et al. [21] proposed weighted mean-field reinforcement learning, modeling pairwise communications between drones as interactions between a central drone and virtual agents abstracted from weighted neighbor aggregations. Li et al. [22] introduced the attention-based intrinsic reward mixture network (AIRMN), featuring an intrinsic reward module designed upon attention mechanisms to enhance cooperative efficacy. Jiang et al. [23] developed graph convolutional reinforcement learning (DGN), where dynamically adaptive graph convolutions capture agent interactions through relation kernels. Facing dynamic topological challenges, contemporary approaches primarily leverage GNN, attentional frameworks, or hybrid architectures to augment collaborative robustness and information utilization efficacy through relation kernel modeling, virtual agent abstraction, or intrinsic reward optimization under topological evolution.

Although these methods have achieved good performance in adapting to dynamic topologies, most of them are only applicable to purely discrete or purely continuous action spaces and lack targeted design for hybrid action spaces with strong coupling between discrete and continuous dimensions. They cannot effectively model the joint impact of dynamic topology changes and hybrid action coupling on policy learning, and it is difficult to maintain stable convergence in hybrid action scenarios with dynamic topologies. In addition, most of these methods only focus on the spatial interaction modeling at the current time step and lack constraints on the temporal consistency of the policy when the topology changes, which makes the attention distribution easily disturbed by the noisy information brought by topology changes, leading to poor generalization of the learned policy.

To sum up, the key barriers of existing research are three-fold: first, the lack of a hybrid action decoupling mechanism that can maintain stability under dynamic topologies; second, the lack of a dynamic interaction modeling method that can adapt to hybrid action spaces; third, the lack of temporal consistency constraints to stabilize policy learning during topology transitions. Most conventional RL benchmarks exclusively accommodate purely discrete or continuous actions—a design choice adopted in previous works primarily because such simple action space problems are easier to model, solve, and verify. Additionally, the core logic of many basic real-world tasks can be temporarily decomposed into independent discrete or continuous action space problems to simplify research, reducing the difficulty of algorithm design, training, and evaluation. This has led to scarce research on MARL algorithms for hybrid action spaces under dynamic topologies. Nevertheless, with the growing demands of real-world multimodal tasks—such as unmanned swarm combat, embodied intelligence, and autonomous driving decision-making—the significance of addressing this research gap is progressively elevating. Unlike the traditional approach of decomposing problems first and solving them separately, designing an end-to-end algorithm directly for hybrid action spaces can fundamentally eliminate error accumulation in intermediate links, achieve global optimization of task effects, greatly simplify the system architecture, reduce research and development costs, and significantly improve the iteration efficiency and scenario generalization ability of the scheme.

Therefore, targeting the above core barriers, this paper proposes the Parametrized Graph Convolution Reinforcement Learning (P-DGN) algorithm. Operating within the actor–critic framework, P-DGN decouples the optimization pathways for hybrid actions, uses multi-head attention to build dynamic relation kernels to adapt to topology changes, and develops temporal relation regularization (TRR) to improve policy consistency across time steps so as to achieve stable and efficient policy learning for MARL in hybrid action spaces under dynamic topologies.

## 3. Parametrized Graph Convolutional Reinforcement Learning

### 3.1. Overview

**Figure 1:**
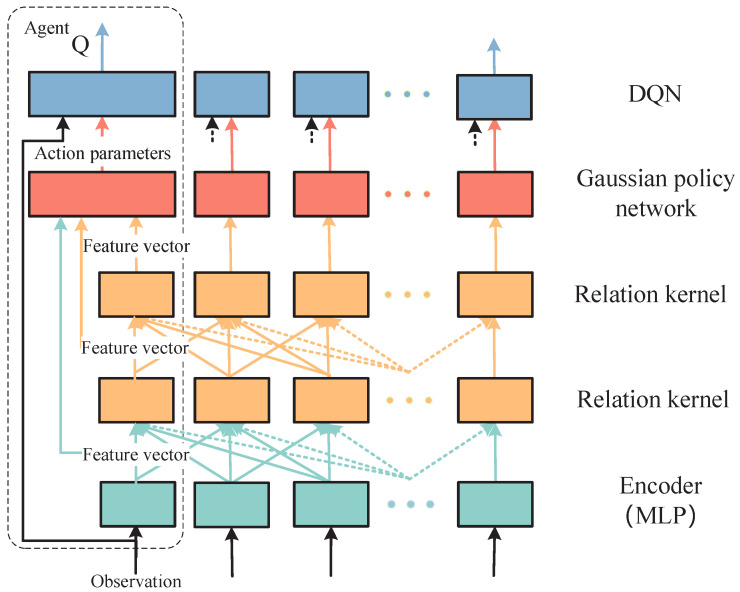
The framework of P-DGN. The P-DGN first receives the observation of each agent and performs feature encoding via a multi-layer perceptron (MLP). It then aggregates the information from neighboring agents through multiple relation kernels and feeds the generated feature vector into the Gaussian policy network via residual connection to output the corresponding action parameters. Finally, the observation and action parameters are jointly input into the deep q-network (DQN) to calculate the Q-value of each hybrid action. Since all agents are homogeneous, the algorithm adopts a parameter-sharing mechanism, where all agents share a unified set of network parameters.

The research focuses on a MARL problem characterized by dynamic observation spaces and hybrid action spaces. While DGN [23] effectively address dynamic observation spaces in highly dynamic multi-agent environments, their action selection mechanism—where actions are chosen based on Q-values output by a Q-network—limits DGN exclusively to discrete action spaces. Inspired by parametrized deep Q-network (P-DQN) [24] for hybrid action spaces processing, we extend DGN to the P-DGN framework, enabling adaptation to hybrid action spaces learning in complex dynamic topologies. The framework of P-DGN is as shown in Figure 1.

For environmental modeling, we represent MAS as a graph: each agent serves as a node, with neighbor sets dynamically determined through distance thresholds. This design stems from two core principles: First, physical proximity among neighboring agents induces stronger interactive influences; Second, global information integration incurs high bandwidth costs and computational complexity [25,26]. Consequently, we create a biologically inspired observation spaces: each agent observes only its seven nearest neighbors’ states [13], constructing distance-sorted observation vectors. This approach optimizes swarm coordination efficiency in noisy environments, reflects an evolutionary balance between group robustness and individual energy consumption, and enables unlimited cooperative potential through convolutional receptive field expansion.

P-DGN shares similarities with communication neural net (CommNet) [27] as both implement centralized strategies via distributed execution, jointly optimizing actions to maximize global expected returns. Their core decomposition manifests through shared global parameters. Within this framework, each agent’s model connects to neighboring nodes via dynamic graph structures, with graph topologies autonomously evolving through real-time agent interactions. Compared to traditional parameter-sharing approaches like DQN, P-DGN innovatively adopts agent-graph-based cooperative sampling—rather than isolated individuals—to explicitly model multi-agent interactions. All agents share identical policy networks, granting P-DGN exceptional scalability. Notably, experiments confirm that despite parameter sharing, P-DGN effectively facilitates complex cooperative strategies, validating its balanced strength in concise modeling and policy expressiveness.

### 3.2. The Actor–Critic Algorithm in P-DGN

In the traditional actor–critic framework, the actor selects actions according to a probability distribution, while the critic evaluates scores based on the actions generated by the actor. The actor subsequently adjusts its action selection probabilities using the critic’s ratings. This cyclical process iteratively optimizes policy decision-making through continuous feedback.

**Figure 2:**
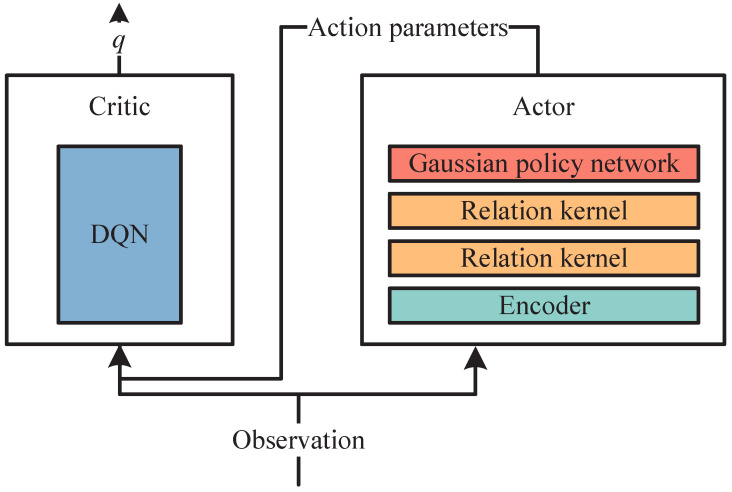
The actor–critic in P-DGN. P-DGN’s actor–critic framework differs from conventional approaches. The network outputs combine continuous parameters from the actor network and discrete actions selected by the critic through Q-value maximization. This addresses the hybrid action spaces requirement where agents simultaneously execute discrete actions and continuous parameters each timestep. These dual components directly correspond to P-DGN’s design: discrete actions are chosen by the critic while continuous parameters are generated by the actor.

The actor–critic in P-DGN is shown in Figure 2.

During P-DGN training, action selection follows an ϵ-greedy strategy. This approach probabilistically balances exploration and exploitation: with probability ϵ, the agent explores by randomly sampling actions regardless of their Q-values; with probability 1−ϵ, it exploits by selecting the action with the highest Q-value. Crucially, ϵ adopts larger values during initial training stages to enhance Q-value exploration across actions while progressively decreasing in later stages to maximize cumulative rewards. Consequently, ϵ is implemented as a monotonically decreasing function throughout training.

P-DGN’s critic employs two neural networks: an online network for action selection and a target network for computing temporal-difference (TD) targets. These networks share identical architectures but maintain distinct parameters. During each learning step, the target network’s parameters undergo soft updates guided by the online network’s parameters, with deliberately constrained update magnitudes. This design significantly enhances learning stability.

### 3.3. Critic: Deep Q-Network

Within the P-DGN framework, the critic network serves as the core value estimation module, adopting a DQN. Its fundamental objective is to dynamically refine state-action value estimates through TD error loss optimization, thus enabling reliable guidance for policy gradient updates in the actor network.

The training phase employs a distributed learning framework with experience replay: at each timestep, the system stores transition tuples $\left(O,A,X,C,R,O^{\prime }\right)$ into a replay buffer, where $O=\left\{o_{1},\ldots ,o_{N}\right\}$ denotes current observation set, $A=\left\{a_{1},\ldots ,a_{N}\right\}$ represents agent action set, $X=\left\{x_{a_{1}},\ldots ,x_{a_{N}}\right\}$ signifies continuous parameters corresponding to discrete actions, $C=\left\{C_{1},\ldots ,C_{N}\right\}$ encodes adjacency matrix set, $R=\left\{r_{1},\ldots ,r_{N}\right\}$ indicates instantaneous reward set, and $O^{\prime }=\left\{{o^{\prime }}_{1},\ldots ,{o^{\prime }}_{N}\right\}$ designates next-state observation set. Concurrent network optimization occurs through random sampling of minibatches of size *S* from this buffer, thus facilitating distributed training, as shown in Equation (1):

(1) $$L_{\mathrm{critic}}\left(\omega \right)=\frac{1}{S}\sum_{S}\frac{1}{N}\sum_{i=1}^{N}{\left[y_{i}-Q\left(o_{i,C},a_{i},x_{a_{i}};\omega \right)\right]}^{2}$$

where $y_{i}=r_{i}+\gamma \max_{a^{\prime }}Q\left(o_{i,C}^{\prime },a^{\prime },x_{a^{\prime }};\omega \right)$ and $o_{i,C}$ represents the observation set within agent *i*’s local perceptual field defined by adjacency matrix *C*, with γ denoting the discount factor. For a given problem, γ is a constant that balances the significance of immediate and future rewards. Its value is determined empirically based on the specific requirement for long-term planning. *N* represents the number of agents. ω represents the parameters of the network. Since agent actions dynamically alter graph structures (e.g., positional shifts causing neighbor relation changes), Q-functions ideally adapt to topological evolution. However, rapid graph variations may induce Q-network oscillation convergence issues. To address this, we introduce topological stability constraint: during loss computation, adjacency matrices *C* remain fixed across two consecutive timesteps. This provides a short-term stable topological learning environment for Q-networks. This strategy decouples the coupling effect between dynamic topologies and Q-value estimation by delaying graph structure updates, significantly enhancing training stability while preserving policy sensitivity to graph evolution.

### 3.4. Actor: Graph Convolutional Policy Network

The graph convolutional policy network integrates three core modules: an observation encoder, convolutional layers, and a Gaussian policy network. The observation encoder utilizes a MLP to encode agent’s local observation into an initial feature vector, mapping raw observations to latent representations. Convolutional layers dynamically aggregate feature information from node and its neighbors via relation kernels, generating contextually fused latent features. The stacking depth directly determines the agents’ receptive field scope—single-layer convolution integrates features within one-hop neighborhoods, while double-layer convolution captures interaction patterns of two-hop nodes through hierarchical abstraction, theoretically supporting unbounded cooperative ranges. Crucially, actual communication remains strictly confined to direct neighbors, ensuring low-bandwidth compatibility in real-world deployments. The Gaussian policy network parameterizes means and variances for continuous actions based on convolved latent features, establishing stochastic policy modeling. Although deep convolutional hierarchies indirectly acquire broad-range information through progressive propagation, each agent communicates exclusively with physically proximal nodes, eliminating global synchronization overheads.

Inspired by DenseNet [28], this study devises a cross-layer feature fusion architecture for each agent: features maps from all network layers undergo channel-wise concatenation, forming a composite representation with multi-scale receptive fields. This concatenated output serves as input to the Gaussian policy network. The design enables effective reuse of observational features at varying abstraction levels—shallow layers capture local interaction patterns while deep layers extract global collaborative relationships. Their complementary integration empowers agents to dynamically synthesize cooperation cues across spatial scales, thereby enhancing representational richness and decisional adaptability in multi-agent coordination strategies.

#### 3.4.1. Relation Kernel

Building upon the relational reinforcement learning (RRL) framework by Zambaldi et al. [29], this study employs multi-head dot-product attention to model agent interactions, replacing conventional convolutional kernels. Specifically, for any agent *i*, we redefine its neighborhood set $B_{i}$ to incorporate the ego agent itself, forming an extended set $B_{+i}=B_{i}\cup \left\{i\right\}$. At each attention head *m*, the interaction weight $\alpha_{ij}^{m}$ between agent *i* and a neighboring agent $j\in B_{+i}$ is computed via a normalized dot-product operation, as formulated in Equation (2):

(2) $$\alpha_{ij}^{m}=\frac{\exp\left[\left(W_{Q}^{m}h_{i}\right)\cdot {\left(W_{K}^{m}h_{j}\right)}^{T}/\sqrt{d}\right]}{\sum_{k\in B_{+i}}\exp\left[\left(W_{Q}^{m}h_{i}\right)\cdot {\left(W_{K}^{m}h_{k}\right)}^{T}/\sqrt{d}\right]}$$

**Figure 3:**
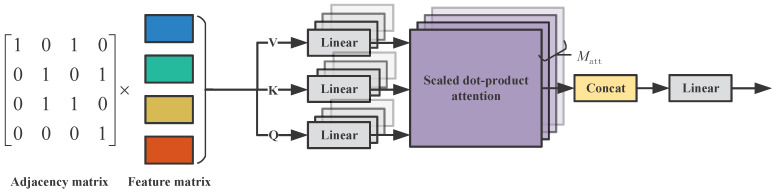
Schematic of the relation kernel. The relation kernel first filters the feature vectors of corresponding neighbor nodes based on the topological relationship defined by the adjacency matrix and then generates the updated node features via the multi-head attention mechanism.

Here, $h_{i}$ represents the input feature vector of agent *i*. $W_{Q}^{m}$ and $W_{K}^{m}$ are the learnable Query and Key projection matrices for head *m*, respectively. The variable *d* denotes the dimensionality of the key vectors, serving as a scaling factor to prevent vanishing gradients in the softmax function. Neighboring information is then aggregated via weighted summation. Subsequently, the outputs from all $M_{\mathrm{att}}$ attention heads are concatenated along the feature dimension and transformed through a single-layer MLP, denoted as σ, with a ReLU activation function. This process, which yields the updated features $h_{i}^{\prime }$, accomplishes convolution-style local interaction modeling as shown in Equation (3):

(3) $${h_{i}}^{\prime }=\sigma \left[\mathrm{concatenate}\left(\sum_{j\in B_{+i}}\alpha_{ij}^{m}W_{V}^{m}h_{j},\forall m\in \left\{1,2,\ldots ,M_{\mathrm{att}}\right\}\right)\right]$$

where $W_{V}^{m}$ is the Value projection matrix of head *m*. A schematic of the relation kernel is shown in Figure 3. This multi-head attention mechanism achieves efficient relational modeling through its distinctive architecture. By employing permutation-invariant attention kernels, this framework guarantees robustness against agent ordering changes in dynamic interactions. Furthermore, it projects features into heterogeneous semantic subspaces via multiple learnable parameter sets ($W_{Q}^{m},W_{K}^{m},W_{V}^{m}$). This enables distinct attention heads to capture diverse interaction patterns, such as spatial proximity and motion trend correlations. Finally, multi-layer stacking progressively extracts features through hierarchical abstraction—shallow layers model local direct relations (e.g., collision avoidance constraints), while deeper layers capture implicit high-order dependencies in group coordination (e.g., task allocation priorities).

#### 3.4.2. Temporal Relation Regularization

In dynamic multi-agent cooperative scenarios, P-DGN employs Temporal Relation Regularization (TRR) to stabilize cooperation relationships across sequential timelines. This method constrains the attention weight distributions generated by relation kernels, ensuring temporal consistency in relational representations. Building on temporal difference learning principles, TRR treats the attention weight distribution at the next timestep t+1 as a stable optimization target. By applying the Kullback–Leibler (KL) divergence to quantify discrepancies between the current and target distributions, the framework minimizes this deviation to construct a regularization constraint.

Let $G_{m}^{\kappa }\left(o_{i,C},\theta \right)$ define the attention weight distribution of agent *i* at the current timestep observation $o_{i,C}$, utilizing network parameters θ, for convolutional layer κ and attention head *m*. Correspondingly, let $o_{i,C}^{\prime }$ denote the observation at the next timestep. The TRR loss term is formalized in Equation (4):

(4) $$L_{\mathrm{KL}}\left(\theta \right)=\lambda \frac{1}{\mathrm{conv}}\sum_{\kappa =1}^{\mathrm{conv}}\frac{1}{M_{\mathrm{att}}}\sum_{m=1}^{M_{\mathrm{att}}}D_{\mathrm{KL}}\left[G_{m}^{\kappa }\left(o_{i,C};\theta \right)\;\|\;G_{m}^{\kappa }\left(o_{i,C}^{\prime };\theta \right)\right]$$

where λ denotes the regularization coefficient balancing the TRR loss, conv represents the total number of convolutional layers, and $M_{\mathrm{att}}$ is the total number of attention heads. This TRR term compels the minimization of KL divergence between attention weight distributions of adjacent timesteps. This mechanism promotes the emergence of resilient cooperation strategies that withstand localized state perturbations. Crucially, this design intrinsically aligns with the delayed reward characteristics of cooperative tasks: achieving most collaborative objectives relies on sustained, stable interaction patterns among agents rather than a simple aggregation of instantaneous reactions.

#### 3.4.3. Gaussian Policy Network

The Gaussian policy network represents a fundamental approach in RL for continuous action spaces problems, parameterizing action stochasticity through Gaussian distributions to enable flexible action exploration and policy optimization in complex control tasks. Specifically, this network generates action distributions with state-dependent mean and variance parameters, progressively approaching optimal policies via gradient-based optimization.

**Figure 4:**
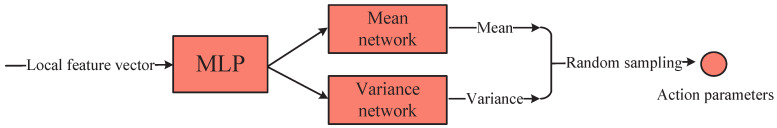
Gaussian policy network structure. The Gaussian policy network typically comprises two subnetworks: a mean network and a variance network. The former employs a MLP to fit nonlinear mappings from states to action expectations, while the latter dynamically regulates exploration intensity. This dual-network configuration ensures sufficient stochasticity during initial training stages, converging toward deterministic actions in later phases.

The structure of the Gaussian policy network is shown in Figure 4.

#### 3.4.4. Loss

The action-value function adopts a decomposed formulation: $Q(o_{i,C,t},a_{i,t},x_{a_{i,t}})$, which estimates the expected long-term cumulative reward of agent *i* when selecting discrete action $a_{i,t}$ with corresponding continuous parameter $x_{a_{i,t}}$, given the local observation $o_{i,C,t}$ (the observation set within agent *i*’s local perceptual field at timestep *t*, defined by adjacency matrix *C*). Based on this definition, the Bellman equation for the action-value function is formalized as Equation (5):

(5) $$Q\left(o_{i,C,t},a_{i,t},x_{a_{i},t}\right)=E[r_{i}+\gamma \max_{a_{i}}\underset{x_{a_{i}}}{\sup}Q\left(o_{i,C,t+1},a_{i},x_{a_{i}}\right)]$$

where E denotes the expectation over the stochastic dynamics of the environment, $r_{i}$ is the instantaneous reward obtained by agent *i* at the current timestep, γ∈(0,1) is the discount factor that balances the weight of immediate and future rewards. The nested max and sup operations correspond to the two-step optimization of our hybrid action space: first, for each candidate discrete action $a_{i}$, we use the supremum operation $\sup_{x_{a_{i}}}$ to find the optimal continuous parameter $x_{a_{i}}$ that maximizes the Q-value in the continuous parameter space (the supremum is used here instead of max because the continuous parameter space is an infinite set, which cannot be exhaustively enumerated to find the maximum value). Then, we use the $\max_{a_{i}}$ operation to select the discrete action with the highest Q-value corresponding to its optimal continuous parameter.

Although direct computation of the supremum in continuous spaces is theoretically intractable, we address this challenge by introducing a parametric policy network to generate near-optimal approximants for $x_{a_{i}}$, enabling achievable and stable optimization. We use a parameterized Q-network $Q(o_{i,C},a_{i},x_{a_{i}};\omega )$ (with trainable parameters ω) to approximate the true action-value function $Q(o_{i,C},a_{i},x_{a_{i}})$, enabling global value estimation across complex hybrid action spaces. Simultaneously, we design a parametric policy network $x_{a}\left(o_{i,C};\theta \right)$ (with trainable parameters θ), which takes the local observation as input and outputs the continuous parameter for each discrete action. The optimization objective of the policy network is to make the Q-value corresponding to the generated continuous parameter as close as possible to the theoretical supremum. Formally, when the Q-network parameters ω are fixed, we seek the optimal policy network parameters θ that satisfy Equation (6):

(6) $$Q(o_{i,C},a_{i},x_{a}\left(o_{i,C};\theta \right);\omega )\approx \underset{x_{a_{i}}}{\sup}Q\left(o_{i,C},a_{i},x_{a_{i}};\omega \right)\;\mathrm{for}\;\mathrm{each}\;a_{i}$$

The loss function of the policy network we design is as Equation (7):

(7) $$L_{\mathrm{actor}}\left(\theta \right)=-\sum_{a}Q\left(o_{i,C},a_{i},x_{a}\left(o_{i,C};\theta \right);\omega \right)+L_{\mathrm{KL}}\left(\theta \right)$$

Building upon this formulation, we implement online weight updates via stochastic gradient methods and two-timescale updating rule [30], grounded in stochastic approximation theory [31]. The core mechanism employs distinct step-size schedules for parameters θ (primary) and ω (auxiliary), where θ’s asymptotic convergence rate attains asymptotically slower decrement. Algorithm stability requires the step-size sequences to satisfy the Robbins–Monro condition [32]. Unlike conventional temporal smoothing methods (e.g., moving averages or recurrent neural network), TRR’s explicit distribution alignment mechanism effectively mitigates attention weight fragmentation caused by neighbor permutations in dynamic topologies, thus delivering robust cooperative solutions in open dynamic scenarios. These methodological choices ultimately culminate in the P-DGN algorithm, whose complete implementation is detailed in Algorithm 1.
**Algorithm 1** Parametrized graph convolutional reinforcement learning approach**Require:** All parameters**Ensure:** θ (Parameters of the actor network), ω (Parameters of the critic network)  1:**for** each *t* **do**  2:      **for** each agent *i* **do**  3:            Calculating action parameters $x_{a_{i}}\leftarrow x_{a}\left(o_{i,C},\theta \right)$  4:            Compute action values with online network $q\leftarrow Q(o_{i,C},a_{i},x_{a_{i}};\omega )$  5:            Based on the *q*, the action is selected with ϵ-greedy strategy $\left(a_{i},x_{a_{i}}\right)$  6:            Execute the action to get $\left(R,O^{\prime }\right)$  7:      **end for**  8:      Put the tuple $\left(O,A,X,C,R,O^{\prime }\right)$ into the replay buffer Γ  9:**end for**10:**for** each epoch **do**11:      a set of $\left(O_{b},A_{b},X_{b},C_{b},R_{b},{O^{\prime }}_{b}\right)$ is obtained by randomly sampling from Γ12:      Calculate $y_{b}$ with the target network $y_{b}\leftarrow r_{b}+\gamma \max_{{a^{\prime }}_{b}}Q\left(o_{b}^{\prime },a_{b}^{\prime },x_{{a^{\prime }}_{b}};\omega \right)$13:      θ and ω in the online network are updated by $L_{actor}\left(\theta \right)$ and $L_{critic}\left(\omega \right)$ with learning rates $\alpha_{actor}$ and $\alpha_{critic}$14:      The soft update coefficient τ is used to update θ and ω in the target network15:**end for**16:**return** θ, ω

## 4. Simulation

### 4.1. Multi-Agent Collaborative Task Allocation and Target Engagement

#### 4.1.1. Environment

**Figure 5:**
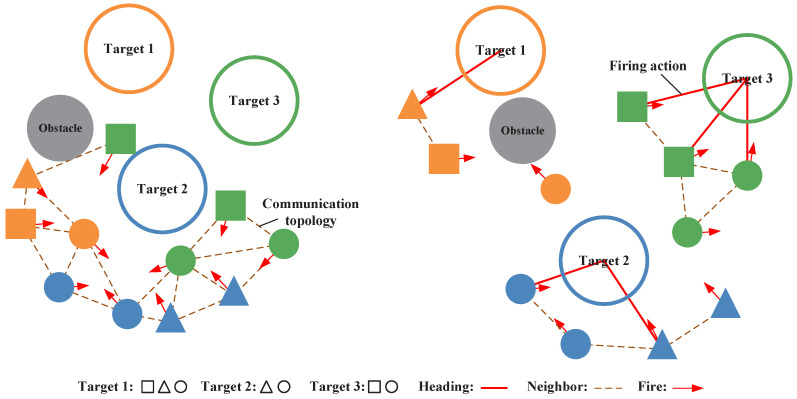
Schematic diagram of the simulation scenario. In the environment, task targets are visualized as three hollow circles in yellow, blue, and green. Agents are represented by solid-colored geometric shapes, where distinct shapes denote differential capability constraints (i.e., variant target engagement authorizations, see legend). Agent color indicates current target selection (color-synchronized with targets). Directional arrows depict heading vectors, while brown dashed lines signify neighbor relationships between agents. During simulations, red solid lines denote fire actions, and gray circles represent obstacles.

The simulation scenario features multi-agent collaborative task allocation (left panel) and target engagement (right panel). Task allocation assigns agents to targets, achieved via the confidence proxy-driven posterior correction and greedy swapping method (CPPC-GS) which dynamically updates agent selections when target counts change. Inspired by [33], target engagement constitutes a navigation-obstacle avoidance and simplified fire-control process: agents evade obstacles and approach designated targets, executing fire commands upon alignment. Successful strikes are determined when projecting a range extension along heading vectors intersects target volumes. The proposed P-DGN algorithm is employed in this phase, training agents to learn cooperative target engagement strategies to accomplish the mission as shown in Algorithm 2 and Figure 5.
**Algorithm 2** Multi-agent collaborative task allocation and target engagement workflow**Require:** All parameters**Ensure:** *A* (Agent action set), *X* (Continuous parameters corresponding to discrete actions)  1:**for** each *t* **do**  2:      **if** $M_{t}\neq M_{t-1}$ **then**  3:            Use the CPPC-GS to reallocate tasks and update *O* with new tasks  4:      **end if**  5:      **for** each agent *i* **do**  6:            Obtain $\left(a_{i},x_{a_{i}}\right)$ via inference using the P−DGN: $\left(a_{i},x_{a_{i}}\right)\leftarrow P\text{-}\mathrm{DGN}\left(o_{i,C},\theta ,\omega \right)$  7:      **end for**  8:**end for**  9:**return** *A*, *X*

During model training and evaluation, obstacle centroids and radii, along with agent initial positions, are uniformly randomized within the bounded map area.

Subsequent experiments will validate algorithm efficacy using point-mass UAV model as benchmark agent. Inspired by [21], muti-agent intrinsic dynamics are characterized as Equation (8):

(8) $$\left\{\begin{array}{c} {\dot{x}}_{{\mathrm{uav}}_{i}}=v_{{\mathrm{uav}}_{i}}\cos\theta_{{\mathrm{uav}}_{i}}\\ {\dot{y}}_{{\mathrm{uav}}_{i}}=v_{{\mathrm{uav}}_{i}}\sin\theta_{{\mathrm{uav}}_{i}}\\ {\dot{v}}_{{\mathrm{uav}}_{i}}=a_{{\mathrm{uav}}_{i}}\\ {\dot{\theta }}_{{\mathrm{uav}}_{i}}=\omega_{{\mathrm{uav}}_{i}} \end{array}\right.\;\;\;\forall i\in \left\{1,2,\ldots ,N\right\}$$

The platform’s state vector ${\left[x_{{\mathrm{uav}}_{i}},y_{{\mathrm{uav}}_{i}},v_{{\mathrm{uav}}_{i}},\theta_{{\mathrm{uav}}_{i}}\right]}^{T}$ comprises position $x_{{\mathrm{uav}}_{i}}$ and $y_{{\mathrm{uav}}_{i}}$, velocity magnitude $v_{{\mathrm{uav}}_{i}}$, and heading angle $\theta_{{\mathrm{uav}}_{i}}$. Control inputs ${\left[a_{{\mathrm{uav}}_{i}},\omega_{{\mathrm{uav}}_{i}}\right]}^{T}$ include acceleration $a_{{\mathrm{uav}}_{i}}$ and heading angular rate $\omega_{{\mathrm{uav}}_{i}}$.

Following discretization, the platform’s kinematic model is formalized as Equation (9):

(9) $$\left\{\begin{array}{c} v_{{\mathrm{uav}}_{i}}^{k+1}=v_{{\mathrm{uav}}_{i}}^{k}+a_{{\mathrm{uav}}_{i}}^{k+1}\cdot \Delta t\\ \theta_{{\mathrm{uav}}_{i}}^{k+1}=\theta_{{\mathrm{uav}}_{i}}^{k}+\omega_{{\mathrm{uav}}_{i}}^{k+1}\cdot \Delta t\\ x_{{\mathrm{uav}}_{i}}^{k+1}=x_{{\mathrm{uav}}_{i}}^{k}+v_{{\mathrm{uav}}_{i}}^{k+1}\cdot \cos\theta_{{\mathrm{uav}}_{i}}^{k+1}\cdot \Delta t\\ y_{{\mathrm{uav}}_{i}}^{k+1}=y_{{\mathrm{uav}}_{i}}^{k}+v_{{\mathrm{uav}}_{i}}^{k+1}\cdot \sin\theta_{{\mathrm{uav}}_{i}}^{k+1}\cdot \Delta t \end{array}\right.\;\;\;\forall i\in \left\{1,2,\ldots ,N\right\}$$

Given a minimum turning radius $R_{\mathrm{uav},\min}$, the platform’s kinematic constraints—including non-holonomic turning limits and inability to hover—are enforced by bounding velocity magnitude $v_{{\mathrm{uav}}_{i}}$, heading angular rate $\omega_{{\mathrm{uav}}_{i}}$, and acceleration magnitude $a_{{\mathrm{uav}}_{i}}$ as Equation (10):

(10) $$\left\{\begin{array}{c} a_{{\mathrm{uav}}_{i}}\leq a_{\mathrm{uav},\max}\\ \omega_{{\mathrm{uav}}_{i}}\leq \omega_{\mathrm{uav},\max}=\frac{v_{{\mathrm{uav}}_{i}}}{R_{\mathrm{uav},\min}}\\ v_{\mathrm{uav},\min}\leq v_{{\mathrm{uav}}_{i}}\leq v_{\mathrm{uav},\max} \end{array}\right.\;\;\;\forall i=1,2,\ldots ,N$$

#### 4.1.2. Design of Observations, Actions and Rewards

The agent’s observation space is a 65-dimensional feature vector, composed of the lateral and longitudinal positions, velocity magnitudes, and physical sizes of the target under engagement, obstacles, other targets, the ego agent itself, and its seven nearest neighbors, constituting a 13 × 5 structure.

The action space consists of three discrete commands: two agent control inputs $a_{{\mathrm{uav}}_{i}}$ and $\omega_{{\mathrm{uav}}_{i}}$, plus a fire command, with each corresponding to continuous parameter ranges [−20, 20], [−5, 5], and [−π/4, π/4], respectively.

The task reward function integrates individual rewards (comprising approach rewards, collision avoidance rewards, and engagement rewards) with team-level rewards, with specific computation and allocation methodologies detailed in the accompanying Table 1.

Δθ represents the angle between the agent’s heading and the line connecting the agent to the target. $p_{\mathrm{task}}$ denotes the positional coordinates of the task target. $D_{\mathrm{dis},ij}=∥p_{i}-p_{j}∥$. *I* serves as an indicator function, assuming a value of 1 when prescribed conditions are satisfied and 0 otherwise. $R_{\mathrm{hit}}$ defines the agent’s hit range radius, while $R_{j}$ denotes the radius associated with *j*. χ constitutes the set encompassing both task targets and environmental obstacles. $\theta_{{\mathrm{azi}}_{i}}$ represents the azimuth angle of agent *i* relative to its designated target. ${\mathrm{target}}_{i}$ is the target that agent *i* hits. For ${\mathrm{target}}_{i}$, parameter $N_{d_{{\mathrm{target}}_{i}}}$ specifies the minimum number of agents. Set $T_{{\mathrm{target}}_{i}}$ comprises all agents assigned to ${\mathrm{target}}_{i}$.

Stage Rewards, encompassing both the orientation and approach rewards, serve as incremental incentives for completing the sequential phases of target engagement. These stage-based rewards translate the global objective into executable local signals, facilitating efficient policy learning through stepwise evaluation. This mechanism accelerates policy optimization by providing immediate performance feedback, mitigating the accumulation of faults over extended trajectories. The dense reward structure actively guides effective exploration while explicitly decomposing complex objectives—a design that enhance both policy interpretability and long-term planning capabilities.

#### 4.1.3. Parameter Settings

The parameter settings used in the simulation are as Table 2:

The hyperparameters in our model were empirically tuned to balance learning stability, sample efficiency, and computational overhead. Specifically, ${\mathrm{episode}}_{\max}$ specifies the total number of training episodes, ensuring the model experiences sufficient state transitions for convergence. epoch denotes the frequency of sampling and training iterations performed within each episode, and *S* defines the dimensional scale of these sampled mini-batches. These parameters were jointly tuned to maximize data reuse from the replay buffer while preventing severe overfitting to recent experiences. Within the ϵ-greedy strategy, parameters $\epsilon_{\mathrm{start}}$, $\epsilon_{\mathrm{decay}}$, and $\epsilon_{\mathrm{end}}$ collectively implement a linearly decaying ϵ schedule. This decay rate is carefully calibrated to encourage broad state-space exploration in the early stages and smoothly transition to the exploitation of the learned policy as training matures. The discount factor γ is set to heavily weight long-term returns, which is essential for delayed-reward cooperative tasks, while τ governs the soft update ratio of the target networks to prevent Q-value oscillation and ensure stable policy evaluation. Additionally, $M_{\mathrm{att}}$ represents the number of attention heads, chosen empirically to capture diverse multi-agent interaction modalities (e.g., spatial proximity, task focus) without incurring excessive computational cost.

Regarding the environment settings, ${\mathrm{step}}_{\max}$ represents the maximum simulation steps, $t_{\mathrm{step}}$ is the simulation step duration, and $t_{\mathrm{sim}}$ is the total simulation time. These are calibrated to provide agents with adequate temporal horizons to complete complex coordination tasks before episode termination. *M* indicates the number of task targets, each possessing HP that decreases by 1 per successful hit, resulting in target destruction when HP reaches zero. Parameters like the agent communication range $R_{\mathrm{com}}$ and task target radius $R_{\mathrm{task}}$ are defined based on the physical constraints of the specific multi-agent scenario to ensure realistic local observability and interaction boundaries.

During training, network parameters undergo Kaiming initialization to mitigate vanishing or exploding gradients in deep layers, while optimization proceeds via the Adam optimizer with adaptive learning rates for robust convergence. The replay buffer capacity, defined by BUFFER_SIZE, is sized large enough to break temporal correlations in trajectory data but constrained to maintain memory efficiency. $\alpha_{\mathrm{critic}}$ and $\alpha_{\mathrm{actor}}$ represent the respective learning rates for the critic and actor networks; the critic’s learning rate is typically set slightly higher to ensure accurate value estimation guides the actor’s policy updates. Finally, the neural architectures were dimensioned based on the complexity of the observation and action spaces to provide sufficient representational capacity without overfitting: The critic network accepts 68-dimensional inputs and generates 3-dimensional outputs through six sequential hidden layers containing 256, 512, 512, 512, 256, and 128 neurons. The encoder processes 65-dimensional inputs to produce 128-dimensional outputs via a single 512-neuron hidden layer; the convolutional layer transforms 128-dimensional inputs into 128-dimensional outputs using a 128-neuron hidden layer; and the Gaussian policy network maps 384-dimensional inputs to 3-dimensional outputs through two hidden layers containing 1024 and 256 neurons, respectively.

Task targets exhibit modified random trajectory dynamics wherein boundary repulsive forces, obstacle repulsive forces, and agent avoidance forces experience linear enhancement within designated buffer zones. For multi-agent coordination, agents employ the CPPC-GS task allocation algorithm integrated with the Olfati–Saber algorithm [34] for collective navigation and obstacle avoidance, with corresponding algorithmic parameters documented in Appendix A.

#### 4.1.4. Training

All experimental trials were conducted on a computing system equipped with an Intel(R) Core(TM) i9-14900KF CPU, 256 GB of RAM, and an NVIDIA GeForce RTX 4090 GPU, operating under the Ubuntu environment. The algorithms were implemented in Python 3.9.16, utilizing the numerical computing capabilities of NumPy 1.23.5 along with the deep learning framework PyTorch 2.0.0 for neural network construction and optimization procedures.

**Figure 6:**
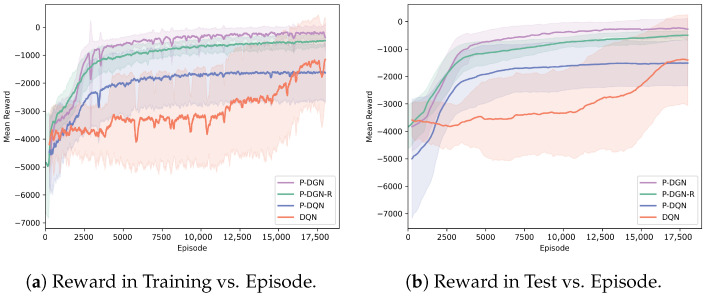
Learning curves in target engagement. For all models, the shaded regions encompass the minimum and maximum values observed across five independent training runs, with solid centerlines representing the corresponding arithmetic means. The **left panel** illustrates learning curves during the training regime, while the **right panel** displays evaluation performance during test (not influenced by the ϵ-greedy strategy).

We conducted parallel training of 4 distinct models—P-DGN, P-DGN-R, P-DQN, and DQN—using identical hyperparameter configurations. As DQN operates within discrete action spaces, a prerequisite discretization procedure was applied to the continuous action parameters prior to its implementation. During the initial 250 episodes, all models exclusively engaged in experience collection without parameter updates (warm-up phase). The temporal evolution of mean reward throughout training and test phases is depicted in Figure 6. Table 3 presents the detailed performance during training in tabular form.

P-DGN achieves significantly superior convergence with considerably higher mean reward than other baselines, demonstrating notably smoother learning trajectories throughout the training process. Although P-DQN exhibits competitive initial learning progress, it ultimately converges to suboptimal reward. Both P-DQN and DQN demonstrate analogous long-term learning efficacy, consistently stagnating at local optima with final performance plateaus substantially below P-DGN’s attainment. This performance divergence stems from the collaborative target engagement requirements: Agents must acquire higher-order neighbor information through dynamically evolving communication topologies to achieve reward maximization during the engagement phase—precisely where P-DGN’s multi-layer stacked relation kernels demonstrate architectural efficacy. The training stability enhancement primarily originates from two algorithmic innovations: topological stability constraint across consecutive timesteps during loss computation and TRR. These mechanisms jointly ensure agents operate within an invariant learning environment, thereby explaining P-DGN’s exceptionally stable convergence behavior.

For the ablation study, we individually ablated the TRR module from the original P-DGN framework (the resulting ablation variant is denoted as P-DGN-R) and trained it under identical experimental settings. However, the variant failed to achieve comparable performance to the full P-DGN model. In simulations, agents controlled by P-DGN-R exhibited hesitation when selecting collaborative partners compared with those governed by the original P-DGN, which led to more collision penalties and impaired the overall collaborative performance.

The core advantage of hybrid action space methods (P-DGN and P-DQN) over discrete action space methods (DQN) resides in their capacity to preserve native action structures and model inter-action dependencies when addressing hybrid action spaces challenges. This foundational capability uniquely enables the circumvention of dimensionality curses, thereby significantly enhancing decision-making precision and operational efficiency.

#### 4.1.5. Evaluation

**Figure 7:**
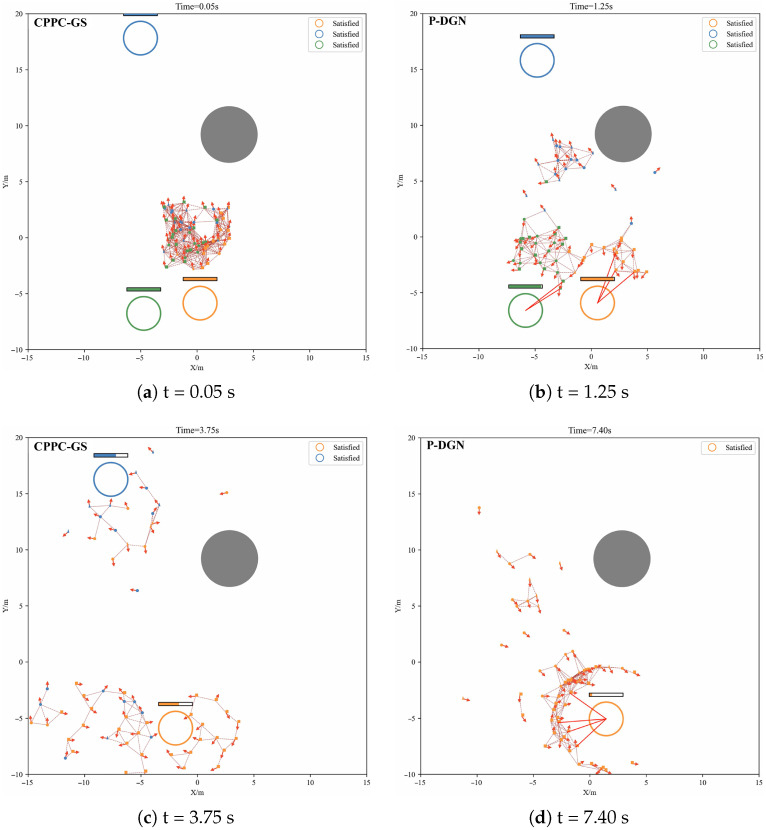
Model evaluation results. Each subfigure incorporates temporal annotation at the top, with the current algorithm phase identified in the upper-left corner (CPPC-GS for task allocation phase; P-DGN for target engagement phase). The legend in the upper-right quadrant quantifies the agent deficit per target—an effectiveness metric for task allocation.

Visualization results of evaluation execution for the trained model within the operational environment are presented in Figure 7. Initial conditions feature all agents spawned within a common region conducting cooperative task allocation (Figure 7a). Following target assignment, agents approach designated targets to execute engagement (Figure 7b). Upon target destruction, agents reconvene centrally to re-initiate the allocation (Figure 7c). Mission completion is achieved when all targets are successfully destroyed (Figure 7d).

The collaborative reward structure—specifically the target hit and coordinated hit rewards—induces emergent encirclement tactic for coordinated engagement (Figure 7b,d) rather than isolated actions. Target movement is constrained to the observable area, while agents unexpectedly develop boundary-exploitation strategy that corral targets toward environmental constraints for efficient destruction.

### 4.2. Modified Simple Spread

**Figure 8:**
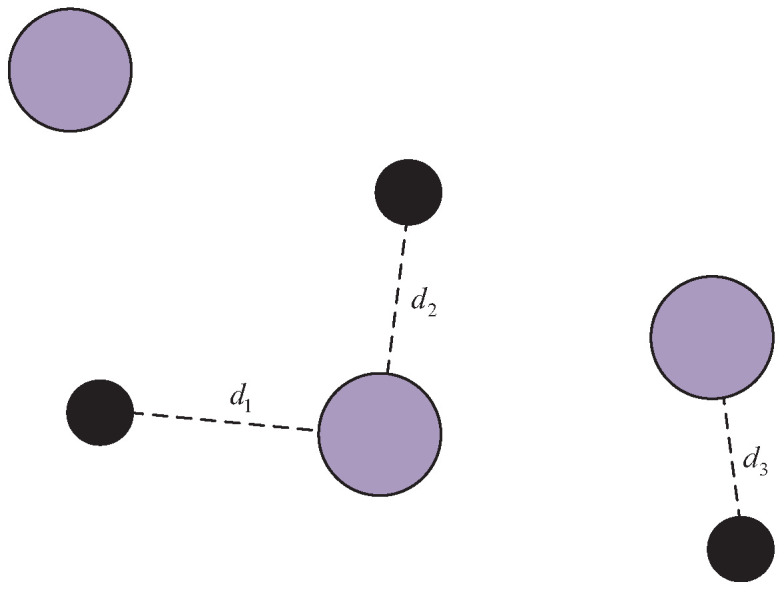
Schematic diagram of the simple spread. Lavender circles represent agents and black circles represent landmarks. The global reward is the sum of the three distances in the figure.

To further investigate the advantages of P-DGN in multi-agent collaboration under general environments, we additionally redeveloped the simple spread environment from multi-agent particle environment (MPE) [35] and conducted comparative experiments. The original version of this environment consists of *N* agents and *N* landmarks. In general, agents are required to learn to cover all landmarks while avoiding collisions with each other, as illustrated in the Figure 8.

More specifically, all agents receive a global reward calculated based on the sum of the shortest distances from each landmark to its nearest agent. Locally, each agent is penalized if it collides with other agents. The relative weights of these reward terms are controlled by the local_ratio parameter. The environment supports switching between discrete and continuous action spaces, as well as adjusting the number of agents, via parameter configuration.

We adapted the aforementioned environment to support hybrid action spaces, local observation spaces, and a modified reward function. Each agent’s observation includes its own velocity and position, the positions of all landmarks, and the positions of the two nearest agents within its communication range (zero-padding is applied if fewer than two agents are within range; we did not use seven neighboring agents in this scenario as the number of agents was set to five). The agent’s action space consists of five discrete actions and their corresponding continuous parameters, namely no_action, move_left, move_right, move_down, and move_up. The parameter represents the magnitude of the force applied in the corresponding direction. Note that the continuous parameter is not required for the first action (no_action).

The reward function of the original environment is more suitable for scenarios with global observation. Under the local observation setting, we incorporated the negative value of the distance between the agent and its nearest landmark into the original reward function, with the mixing ratio controlled by parameters $w_{\mathrm{base}}$ and $w_{\mathrm{local}}$. The parameter settings of the environment are listed in the Table 4.

The initialization of the networks and the optimizer settings are identical to those in the previous experiment. The architecture of each neural network is specified as follows: the critic network accepts 23-dimensional inputs and generates 5-dimensional outputs through 3 sequential hidden layers containing 256, 128, and 256 neurons. The encoder processes 18-dimensional inputs to produce 64-dimensional outputs via a single 32-neuron hidden layer; the convolutional layer transforms 64-dimensional inputs into 64-dimensional outputs using a 64-neuron hidden layer; and the Gaussian policy network maps 192-dimensional inputs to 5-dimensional outputs through 2 hidden layers containing 1024 and 256 neurons, respectively.

**Figure 9:**
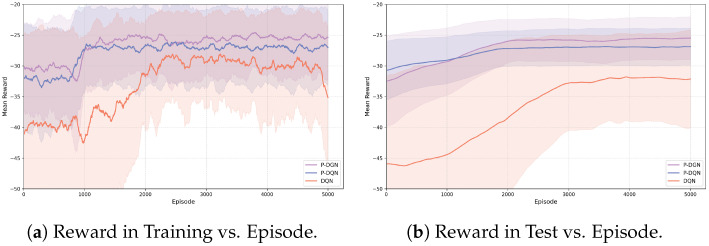
Learning curves in simple spread.

The training environment is also identical to that of the previous experiment. The warm-up phase in this experiment lasts for 800 episodes. The temporal evolution of mean reward throughout training and test phases is depicted in Figure 9. Table 5 presents the detailed performance during training in tabular form.

The simple spread environment emphasizes inter-agent collaboration, requiring agents to cover all landmarks to maximize the collective reward. Under the local observation setting, it is particularly critical for each agent to identify which other agents it should focus on (e.g., agents that have already covered nearby landmarks). This explains why P-DGN outperforms P-DQN without the relation kernel and TRR. In addition, the continuous action parameters enable agents controlled by P-DGN to better decelerate and stop when approaching landmarks, as observed in our simulations. This is another key reason for the superior performance of P-DGN over DQN.

## 5. Conclusions

This paper addresses core challenges in hybrid action spaces MARL within dynamic topologies by proposing P-DGN. The method synthesizes the actor–critic framework, graph convolutional network, and attention mechanisms to effectively resolve collaborative optimization difficulties in hybrid action spaces through decoupled optimization pathways. The critic network employs DQN to evaluate discrete action Q-values while following topological stability constraint across consecutive timesteps, thereby mitigating training oscillations induced by topological dynamics. The actor network constructs relation kernels via multi-head attention mechanisms, incorporates TRR to enforce cross-timestep policy consistency, and generates continuous action parameters through Gaussian policy network. This dual-path architecture establishes a unified theoretical framework for hybrid action spaces collaboration in dynamically evolving topologies.

In two multi-agent cooperation scenarios, P-DGN demonstrates marked superiority over baseline algorithms P-DQN and DQN. Specifically, it exhibits enhanced training convergence speed and stability, achieving higher mean reward with smoother learning trajectories. The architecture’s multi-layer graph convolutional framework facilitates the emergence of sophisticated tactical behaviors—such as encirclement tactic and boundary-exploitation strategy—under dense reward schemes. This capability stems from relation kernels that enable agents to indirectly acquire spatially extensive cooperative intelligence through high-order feature extraction.

Beyond the verified performance advantages in simulation, this work delivers multi-faceted benefits to relevant research and engineering communities.

First, it fills the long-standing research gap of MARL for hybrid action spaces under dynamic topologies. The proposed dual-path actor–critic framework solves the core bottleneck of action decoupling failure and training oscillation under topology changes, while the lightweight TRR and topological stability constraint can be flexibly migrated to various MARL architectures, providing reusable technical components for subsequent research.

Second, this work promotes the cross-integration of biomimetic intelligence and MARL. We verify that the starling-inspired seven-nearest-neighbor interaction strategy improves swarm robustness with low computational cost, providing biologically plausible inspiration for algorithm design and a verifiable framework for biological swarm research.

Finally, it provides an implementable end-to-end decision-making solution for unmanned swarm engineering, promoting the practical deployment of MARL in dynamic open scenarios.

To clarify the strengths and application boundaries of our P-DGN method, we conduct a conceptual comparison with typical meta-heuristic algorithms (genetic algorithm, particle swarm optimization, etc.) across four core dimensions: solution quality, convergence speed, computational cost, and stability.

In terms of solution quality, meta-heuristics only optimize single-step immediate rewards and easily fall into local optima in hybrid discrete-continuous action spaces, while P-DGN is designed for sequential tasks and optimizes long-term global returns of multi-agent systems. For convergence and computational cost, meta-heuristics suffer from exponentially rising overhead with growing agent count and require full research for dynamic environment changes, while P-DGN scales smoothly via parameter-sharing. For stability, meta-heuristics have high output variance and poor constraint compliance, while P-DGN shows stable, reproducible and robust performance.

P-DGN is superior for dynamic multi-agent tasks with real-time requirements, while meta-heuristics fit small-scale static optimization scenarios. We will conduct quantitative experimental comparisons in future work.

## Figures and Tables

**Table 1:** The computation and allocation of rewards in target engagement. The reward structure incorporates several components: The approach rewards incentivize agents to orient their heading toward the task target while penalizing deviation. The collision avoidance rewards promote maintenance of safe distances from other agents, targets, and obstacles. The engagement reward encourages target engagement from diverse angular positions while penalizing fratricides or missed targets. The team-level reward fosters collaborative behavior by incentivizing agents to assist peers during target engagements, thereby enhancing coordination within the MAS.

Object	Type	Name	Formula
Individual	Approach	Orientation Reward	$r_{\mathrm{to},i}=\left\{\begin{array}{cc} 0.2, & \Delta \theta_{i}<\frac{\pi }{4}\\ 0.2\times \left[1-\frac{4}{3\pi }\left(\Delta \theta_{i}-\frac{\pi }{4}\right)\right], & \frac{\pi }{4}\leq \Delta \theta_{i}\leq \pi \end{array}\right.$
		Approach Reward	$r_{\mathrm{app},i}=-0.5\times D_{\mathrm{dis},{\mathrm{uav}}_{i},\mathrm{task}}$
	Collision Avoidance	Collision Avoidance Reward Between UAVs	$r_{\mathrm{dec},i}=-\sum_{j}\left(1-D_{\mathrm{dis},{\mathrm{uav}}_{i}{\mathrm{uav}}_{j}}\right)\cdot I\left(D_{\mathrm{dis},{\mathrm{uav}}_{i}{\mathrm{uav}}_{j}}\leq 1\right),j\in B_{i}$
		Collision Avoidance Reward with Targets/Obstacles	$r_{\mathrm{clear},i}=\left\{\begin{array}{cc} -5\sum_{j}\frac{R_{\mathrm{hit}}+R_{j}-2D_{\mathrm{dis},{\mathrm{uav}}_{i},j}}{R_{\mathrm{hit}}-R_{j}}, & D_{\mathrm{dis},{\mathrm{uav}}_{i},j}\in \left[R_{j},\frac{R_{\mathrm{hit}}+R_{j}}{2}\right]\\ -5, & D_{\mathrm{dis},{\mathrm{uav}}_{i},j}\in \left(0,R_{j}\right) \end{array}\right.j\in \chi$
	Engagement	Target Hit Reward	$r_{{\mathrm{hit}}_{r},i}=2\sum_{j}\frac{1-\cos\left(\theta_{{\mathrm{azi}}_{i}}-\theta_{{\mathrm{azi}}_{j}}\right)}{N_{d_{{\mathrm{target}}_{j}}}-1},\;j\in T_{{\mathrm{target}}_{j}}$
		Fratricide Reward	$r_{{\mathrm{hit}}_{w},i}=-10$
		Target Miss Reward	$r_{{\mathrm{hit}}_{n},i}=-2$
Team Level	Engagement	Coordinated Hit Reward	$r_{{\mathrm{hit}}_{c},i}=\frac{2}{N_{d_{{\mathrm{target}}_{i}}}}\sum_{j}I\left(r_{{\mathrm{hit}}_{r},j}>0\right),j\in B_{i}$

**Table 2:** Algorithm and simulation parameter settings in target engagement.

Type	Name	Value	Type	Name	Value
Algorithm	${\mathrm{episode}}_{\max}$	18,000	Simulation	${\mathrm{step}}_{\max}$	400
	epoch	5		$t_{\mathrm{step}}$	0.05 s
	*S*	512		$t_{\mathrm{sim}}$	20 s
	BUFFER_SIZE	$10^{5}$		*N*	60
	$\alpha_{\mathrm{critic}}$	$5\times 10^{-3}$		$N_{d}$	[10, 15, 20]
	$\alpha_{\mathrm{actor}}$	$5\times 10^{-4}$		*M*	3
	$\epsilon_{\mathrm{start}}$	1		HP	[500, 200, 200]
	$\epsilon_{\mathrm{decay}}$	$1\times 10^{-4}$		$R_{\mathrm{com}}$	3
	$\epsilon_{\mathrm{end}}$	0.1		$R_{\mathrm{task}}$	1.5
	γ	0.9		$R_{\mathrm{hit}}$	5
	τ	0.01		$v_{\mathrm{uav},\min}$	5 m/s
	λ	0.03		$v_{\mathrm{uav},\max}$	10 m/s
	$M_{\mathrm{att}}$	8		$R_{\mathrm{uav},\min}$	0.2 m

**Table 3:** Performance comparison in target engagement. In order to evaluate the final convergence performance of different algorithms on the test set, a Kruskal–Wallis H test was performed on the last episode reward of P-DGN, P-DGN-R, P-DQN, and conventional DQN algorithms. The results show that there is a statistically significant difference between the final rewards of the three algorithms.

	P-DGN	P-DGN-R	P-DQN	DQN
mean reward	−276.81	−498.10	−1512.91	−1401.83
standard deviation	47.96	82.41	671.16	1348.70
best reward	−215.62	−406.17	−390.05	−173.96
median reward	−281.91	−494.84	−1452.45	−968.22
worst reward	−333.35	−623.81	−2214.00	−4029.81

**Table 4:** Algorithm and simulation parameter settings in simple spread.

Type	Name	Value	Type	Name	Value
Algorithm	${\mathrm{episode}}_{\max}$	5000	Simulation	${\mathrm{step}}_{\max}$	25
	epoch	5		local_ratio	0.2
	*S*	512		*N*	5
	BUFFER_SIZE	$10^{5}$		$R_{\mathrm{com}}$	0.5
	$\alpha_{\mathrm{critic}}$	$1\times 10^{-2}$		$R_{\mathrm{agent}}$	0.05
	$\alpha_{\mathrm{actor}}$	$1\times 10^{-3}$		$R_{\mathrm{mark}}$	0.1
	$\epsilon_{\mathrm{start}}$	1		$w_{\mathrm{base}}$	0.4
	$\epsilon_{\mathrm{decay}}$	$2\times 10^{-4}$		$w_{\mathrm{local}}$	0.6
	$\epsilon_{\mathrm{end}}$	0.1			
	γ	0.9			
	τ	0.01			
	λ	0.03			
	$M_{\mathrm{att}}$	8			

**Table 5:** Performance comparison in simple spread. In order to evaluate the final convergence performance of different algorithms on the test set, a Kruskal–Wallis H test was performed on the last episode reward of P-DGN, P-DQN, and conventional DQN algorithms. The results show that there is a statistically significant difference between the final rewards of the three algorithms.

	P-DGN	P-DQN	DQN
mean reward	−25.49	−26.88	−32.15
standard deviation	1.69	0.75	3.35
best reward	−22.79	−26.09	−29.68
median reward	−25.85	−26.78	−30.81
worst reward	−27.29	−28.03	−38.65

## Data Availability

The original contributions presented in this study are included in the article. Further inquiries can be directed to the corresponding author.

## Appendix A. Other Algorithm Parameters

When using CPPC-GS in the simulation chapter, the relevant parameters selected are as follows in Table A1:

**Table A1:** The parameters of CPPC-GS. max_iter is the maximum number of iterations of the algorithm. The range of α is related to the scale of the update amount. The recommended range for α is 10 to $10^{4}$, based on the scale of the parameters for common task allocation problems. δ is a bias added to prevent the core formula of the algorithm from having a zero denominator.

Name	Value
max_iter	30
α	200
δ	$1\times 10^{-8}$

When using Olfati–Saber in the simulation chapter, the relevant parameters selected are as follows in Table A2:

**Table A2:** The parameters of Olfati-Saber. $c_{1}^{\alpha }$, $c_{2}^{\alpha }$, and *h* are hyperparameters that control cluster flocking. $c_{1}^{\beta }$, $c_{2}^{\beta }$, and $d_{s}$ are hyperparameters that control cluster obstacle avoidance. $c_{1}^{\gamma }$ and $c_{2}^{\gamma }$ are hyperparameters that control cluster navigation. ϵ is the σ norm hyperparameter, $d_{\min}$ is the minimum distance between UAVs, $R_{\mathrm{agent}}$ is the physical radius of the agent.

Name	Value	Name	Value
$c_{1}^{\alpha }$	1000	*h*	0.5
$c_{2}^{\alpha }$	63.25	$d_{s}$	0.4
$c_{1}^{\beta }$	800	ϵ	2
$c_{2}^{\beta }$	56.57	$d_{\min}$	0.7
$c_{1}^{\gamma }$	4000	$R_{\mathrm{agent}}$	0.1
$c_{2}^{\gamma }$	126.49		

## References

[1] Li J., Yang Y., He Z., Wu H., Zhao C., Hwang K.S. (2025). Investigating Primacy Bias in Multi-Agent Reinforcement Learning: An Empirical Study. IEEE Trans. Artif. Intell..

[2] Han J., Yan Y., Zhang B. (2025). Towards Efficient Multi-UAV Air Combat: An Intention Inference and Sparse Transmission Based Multi-Agent Reinforcement Learning Algorithm. IEEE Trans. Artif. Intell..

[3] Zhao F., He X., Wang L. (2020). A two-stage cooperative evolutionary algorithm with problem-specific knowledge for energy-efficient scheduling of no-wait flow-shop problem. IEEE Trans. Cybern..

[4] Kucukoglu I., Dewil R., Cattrysse D. (2021). The electric vehicle routing problem and its variations: A literature review. Comput. Ind. Eng..

[5] Hu G. (2024). Scalable learning for multiagent route planning: Adapting to diverse task scales. IEEE Trans. Artif. Intell..

[6] Dong Q., Wu Z., Lu J., Sun F., Wang J., Yang Y., Shang X. (2022). Existence and practice of gaming: Thoughts on the development of multi-agent system gaming. Front. Inf. Technol. Electron. Eng..

[7] Yang L., Li X., Sun M., Sun C. (2023). Hybrid policy-based reinforcement learning of adaptive energy management for the Energy transmission-constrained island group. IEEE Trans. Ind. Inform..

[8] Zhang M., Chen K., Zhu J. (2023). An efficient planning method based on deep reinforcement learning with hybrid actions for autonomous driving on highway. Int. J. Mach. Learn. Cybern..

[9] Wang S., Wang Z., Jiang R., Zhu F., Yan R., Shang Y. (2024). A multi-agent reinforcement learning-based longitudinal and lateral control of CAVs to improve traffic efficiency in a mandatory lane change scenario. Transp. Res. Part C Emerg. Technol..

[10] Zeng Z., Dong C., Wu I.J., Zhu X., Zhang L. (2024). Optimal UAV Swarm Reconstruction Strategy Based on Minimum Cost Maximum Flow Algorithm. 2024 IEEE Wireless Communications and Networking Conference (WCNC).

[11] Xiao B., Li R., Wang F., Peng C., Wu J., Zhao Z., Zhang H. (2023). Stochastic graph neural network-based value decomposition for marl in internet of vehicles. IEEE Trans. Veh. Technol..

[12] Yang C., Yang G., Chen H., Zhang J. (2023). Explicitly Learning Policy Under Partial Observability in Multiagent Reinforcement Learning. 2023 International Joint Conference on Neural Networks (IJCNN).

[13] Young G.F., Scardovi L., Cavagna A., Giardina I., Leonard N.E. (2013). Starling flock networks manage uncertainty in consensus at low cost. PLoS Comput. Biol..

[14] Xie Q., Wang Z., Fang Y., Li Y. (2025). MABQN: Multi-agent reinforcement learning algorithm with discrete policy. Neurocomputing.

[15] Tian S., Yang M., Xiong R., He X., Rajasegarar S. (2024). A sequential multi-agent reinforcement learning framework for different action spaces. Expert Syst. Appl..

[16] Hua H., Zhao R., Wen G., Wu K. (2023). A further exploration of deep multi-agent reinforcement learning with hybrid action space. Artificial Neural Networks and Machine Learning—ICANN 2023.

[17] Li W., Wang X., Jin B., Luo D., Zha H. (2021). Structured cooperative reinforcement learning with time-varying composite action space. IEEE Trans. Pattern Anal. Mach. Intell..

[18] Li M., Wan X., Yan M., Wu J., He H. (2024). Attentive hybrid reinforcement learning-based eco-driving strategy for connected vehicles with hybrid action spaces and surrounding vehicles attention. Energy Convers. Manag..

[19] Bernárdez G., Suárez-Varela J., López A., Wu B., Xiao S., Cheng X., Barlet-Ros P., Cabellos-Aparicio A. (2021). Is machine learning ready for traffic engineering optimization?. 2021 IEEE 29th International Conference on Network Protocols (ICNP).

[20] Ding S., Du W., Ding L., Zhang J., Guo L., An B. (2023). Robust multi-agent communication with graph information bottleneck optimization. IEEE Trans. Pattern Anal. Mach. Intell..

[21] Wang B., Li S., Gao X., Xie T. (2023). Weighted mean field reinforcement learning for large-scale UAV swarm confrontation. Appl. Intell..

[22] Li W., Liu W., Shao S., Huang S., Song A. (2023). Attention-based intrinsic reward mixing network for credit assignment in multi-agent reinforcement learning. IEEE Trans. Games.

[23] Jiang J., Dun C., Huang T., Lu Z. (2018). Graph Convolutional Reinforcement Learning. arXiv.

[24] Xiong J., Wang Q., Yang Z., Sun P., Han L., Zheng Y., Fu H., Zhang T., Liu J., Liu H. (2018). Parametrized deep q-networks learning: Reinforcement learning with discrete-continuous hybrid action space. arXiv.

[25] Tan M. Multi-agent reinforcement learning: Independent vs. cooperative agents. Proceedings of the Tenth International Conference on Machine Learning.

[26] Jiang J., Lu Z. (2018). Learning attentional communication for multi-agent cooperation. Advances in Neural Information Processing Systems 31 (NeurIPS 2018).

[27] Sukhbaatar S., Fergus R. (2016). Learning multiagent communication with backpropagation. Advances in Neural Information Processing Systems 29 (NIPS 2016).

[28] Huang G., Liu Z., Van Der Maaten L., Weinberger K.Q. Densely connected convolutional networks. Proceedings of the IEEE Conference on Computer Vision and Pattern Recognition.

[29] Zambaldi V., Raposo D., Santoro A., Bapst V., Li Y., Babuschkin I., Tuyls K., Reichert D., Lillicrap T., Lockhart E. (2018). Relational deep reinforcement learning. arXiv.

[30] Borkar V.S. (1997). Stochastic approximation with two time scales. Syst. Control Lett..

[31] Kushner H., Yin G. (2006). Stochastic Approximation and Recursive Algorithms and Applications. Stochastic Modelling and Applied Probability.

[32] Robbins H., Monro S. (1951). A stochastic approximation method. Ann. Math. Stat..

[33] Gao H., Cai Y., Cai H., Lu H., Lu J. (2022). Performance Evaluation of Multiagent Reinforcement Learning Based Training Methods for Swarm Fighting. Wirel. Commun. Mob. Comput..

[34] Olfati-Saber R. (2006). Flocking for multi-agent dynamic systems: Algorithms and theory. IEEE Trans. Autom. Control.

[35] Lowe R., Wu Y., Tamar A., Harb J., Abbeel P., Mordatch I. (2017). Multi-Agent actor–critic for Mixed Cooperative-Competitive Environments. Advances in Neural Information Processing Systems 30 (NIPS 2017).

